# Paravertebral abscess and bloodstream infection caused by *Burkholderia pseudomallei* after acupuncture: a case report

**DOI:** 10.1186/s12906-022-03563-8

**Published:** 2022-03-31

**Authors:** Lingyan Xiao, Tong Zhou, Jun Chen, Yanqing Hu, Yishan Zheng

**Affiliations:** Department of Intensive Care Unit, the Second Hospital of Nanjing, Nanjing University of Chinese Medicine, 1-1 Zhongfu Road, Nanjing, 210003 China

**Keywords:** Melioidosis, *Burkholderia pseudomallei*, Acupuncture, Paravertebral abscess, Bloodstream infection, Urinary tract infection, Case report

## Abstract

**Background:**

Various pathogenic bacterial infections caused by acupuncture have raised widespread concern, but paravertebral abscesses and bloodstream infections of *Burkholderia pseudomallei* (B.pseudomallei) after acupuncture have not been reported.

**Case presentation:**

A 49-year-old man was admitted to hospital with recurrent back pain and fever for 1 month, along with the finding of undiagnosed diabetes. He was considered to have tuberculosis because of unrelieved high fever and pulmonary nodules. Bilateral blood culture suggested B.pseudomallei infection, MRI of the lumbar spine suggested paravertebral abscess, and the final diagnosis was paravertebral abscess and bloodstream infection after acupuncture combined with migrating lung infection. He was discharged after abscess debridement and intensive anti-infective therapy, but no further oral antibiotics were administered because of his poor adherence. More than 5 months later, he was readmitted with the urine culture findings of B.pseudomallei. No other abscess formation was observed and he received oral antibiotics for more than 3 months without recurrence.

**Conclusions:**

Acupuncture may lead to B.pseudomallei infection in high-risk groups, and inadequate treatment can lead to recurrent infections.

## Background

Acupuncture is a popular complementary medical technique, particularly in Asian countries, used primarily for the treatment of low back pain. It is a relatively safe method, but significant complications such as blood flow and local tissue infection are still reported, including suppurative arthritis, erysipelas, necrotizing fasciitis, and paravertebral abscess [1]. *Staphylococcus aureus* is the most common cause of infection, including methicillin-resistant *Staphylococcus aureus* (MRSA) infections, as well as cases of Pseudomonas, *Escherichia coli*, Listeria and others [2]. To date, no cases of bloodstream B.pseudomallei infection have been reported following acupuncture.

B.pseudomallei is a conditional pathogen that causes Melioidosis, a zoonotic disease that is generally concentrated between the tropics and subtropics, and is severely underreported in the 45 countries where it is known to be endemic, while in another 34 countries where it has never been reported, Melioidosis is probably endemic [3]. B.pseudomallei infections can present with sepsis, liver abscesses, lung abscesses, soft tissue infections and other manifestations. We hereby report a case of paravertebral abscess and bloodstream infection due to B.pseudomallei infection following acupuncture in a sporadic case with previously undiagnosed diabetes.

## Case presentation

A 49-year-old man presented to our hospital with recurrent intermittent low fever for over a month and high fever for a week. He had a history of HBsAg, HBeAb and HBcAb test positive, and lumbar disc herniation. He told us that he had been experiencing recurrent intermittent low fever for the past month and that his self-treatment with antibiotics was ineffective. One week ago, he developed a high fever in the afternoon with a body temperature as high as 39.9–40.3 °C. He underwent CT scans in the local hospital suggesting bilateral pleural effusion and scattered nodules in both lungs, some close to the subpleural, bronchial wall thickening in the upper lobe of the right lung, patchy nodular shadows along the bronchus and bilateral pleural effusion. After receiving cefoperazone and sulbactam for treatment, his body temperature failed to drop. The possibility of tuberculosis was considered, and he came to our tuberculosis unit for treatment.

On admission, the patient had enhanced body temperature(39.30 °C), a heart rate of 126b.p.m., a respiratory rate of 23 breaths/min and blood pressure of 157/90 mmHg at physical examination. He was listless of spirit, with obvious hyperemia in the skin of the face, neck and chest; Fine wet rales can be heard in the left lung, and breathing sounds in both lower lungs were low, especially on the right side. Combined with imaging analysis, he was diagnosed with tuberculosis and received initial anti-tuberculosis therapy as well as hepatoprotection. Several obvious abnormal infection indicators were observed the next day, including white blood cell count 8.13*10^9^/L, procalcitonin 0.67 ng/mL, and C reaction protein 181 mg/L. HbA1c was significantly higher, with fasting plasma glucose above normal range. Blood gas analysis of the patient indicated hypoxemia (PH 7.49, PO_2_ 70 mmHg, PCO_2_ 34 mmHg, oxygenation index 140 mmHg). Meanwhile blood culture drawn upon admission at the local hospital showed bilateral aerobacteria: B.pseudomallei infection.

**Fig. 1:**
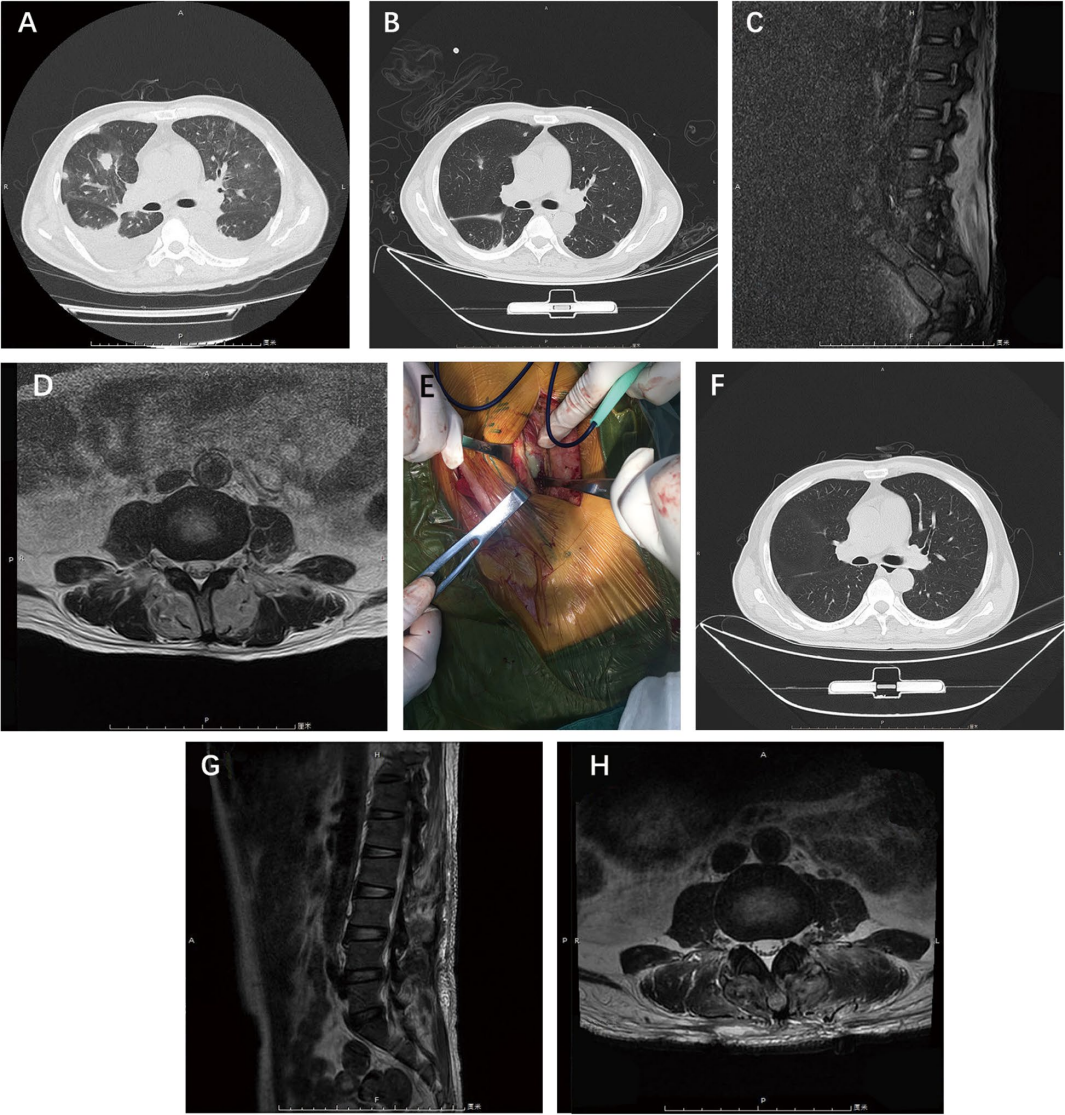
**A**, **B** and **C**, **D**: chest CT and MRI of the lumbar spine before surgery. **F** and **G**, **H**: after surgery. **E**: Operative field

Due to exacerbation of asthma, the patient was given high-flow oxygen therapy and thoracentesis drainage. The breathing condition improved after 2 days. All laboratory data were negative for tuberculosis but the sputum culture suggested candida albictica infection, and reexamination of chest CT showed ground glass shadow around the original lesion and pulmonary interstitial edema, scattered nodules in both lungs unaltered. Consistent with standard care for uncontrolled fever in the presence of undiagnosed diabetes, broad spectrum antibiotics were initiated pending blood culture results. Once the admission blood culture results came back as B.pseudomallei infection, the same as the earlier result, the diagnosis of bloodstream infection of B.pseudomallei and migratory pulmonary nodules was considered and the patient was given treated with meropenem. The question was, where did the bloodstream infection come from? The patient continued to complain of low back pain repeatedly, and MRI of the lumbar spine was arranged, which showed large patches of long T1 and long T2 signal in the subcutaneous soft tissue of the lumbar spine with high signal in the compression lipid phase (Fig. 1).

After further investigation of the medical history, the patient said he had received lumbar acupuncture treatment in the local clinics one week prior to the onset of the disease. Therefore, we inferred that the acupuncture had led to the development of a paravertebral abscess, which eventually entered the blood and caused bloodstream infection, and that multiple migrating lesions were formed in the lungs. The patient then underwent surgical treatment to remove the abscess from the paravertebral area.

**Fig. 2:**
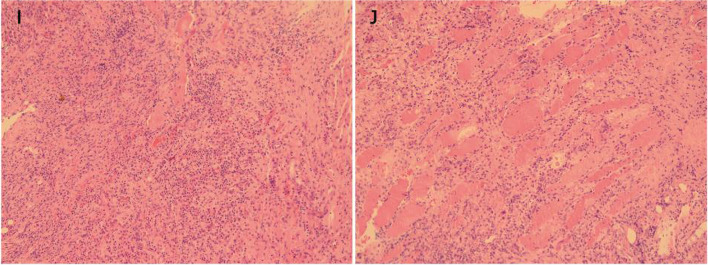
**I**, **J**: Postoperative pathological pictures

Surgeons made two longitudinal incisions along the median incision in the lower lumbar region, and incised the skin, subcutaneous tissue and muscle layer by layer and saw little purulent fluid, a large amount of inflammatory granulation existed inside the spine muscles with unclear boundary (Fig. 1). They completely excised the granulomatous lesions and pus for pathological examination and culture, and then flushed the wound with a large amount of physiological saline, set built-in drainage tube wound irrigation. Postoperative pathology showed large proliferative granulation tissue with massive acute inflammatory cell infiltration, acid fast staining (−)(Fig. 2). Anti-infective treatment with meropenem was continued. Postoperative pus culture remained B.pseudomallei, which was meropenem with blood culture results. After two weeks of continuous anti-infective treatment with meropenem, the patient’s infection indicators normalized, the MRI of the lumbar spine was reviewed and the patient was discharged from hospital (Fig. 1). It was a pity that oral antibiotics were terminated due to his poor adherence.

However, the patient was readmitted five and a half months after discharge for urinary culture suggesting B.pseudomallei. Admission vital signs were stable and no fever was found. Blood glucose tests suggested poor recent blood glucose control (HbA1c 6.5%, reference value 4.0–6.5%; Fructosamine 315 umol/L, reference value 205-285umol/L). Blood routine examination showed WBC 7.02*10^9^/L, N 76.3%, CRP 66.35 mg/L, urine routine examination showed protein 1+, white blood cell 2+, urine ketone body (+−), microscopic examination of 665 white blood cells /uL, microscopic examination of 15 red blood cells /uL, and detection of B.pseudomallei growth. Chest CT was normal. According to previous drug sensitivity, he was given meropenem for anti-infection treatment for 12 days, and his blood glucose was controlled for symptomatic supportive treatment. After the infection control, reexamination of urine routine, urine culture and blood culture showed no obvious abnormality, then the patient was discharged and given oral levofloxacin for anti-infection treatment. The patient was followed up after three months of oral levofloxacin and was clinically cured with no abscess formation elsewhere.

## Discussion and conclusions

We hereby report a case of severe infection with B.pseudomallei after acupuncture. Since B.pseudomallei can survive in water and soil for a long time, we speculate that although the acupuncturist had sterilized the patient’s skin prior to acupuncture, the needles may had been carelessly exposed to tap water. The purpose of our case report is to raise awareness of the need for aseptic technique. Deep infections and bloodstream infections, like the one we report here, are perfect examples of the unfortunate consequences of a failure to maintain such technique.

Factors associated with B.pseudomallei infection include environmental exposure, weather conditions, host’s immune status, and post-exposure immune responses in healthy populations [4]. Diabetes is the most important predisposing factor, seen in more than half of the population, especially type 1 diabetes, followed by alcoholism, chronic lung disease and chronic kidney disease, and long-term use of steroids and immuno-suppression may also lead to the infection. However, a significant number of people (about 20% of adults) have no identifiable risk factors [5]. In our case, the patient complained of no history of diabetes, but the HbA1c was as high as 13.1% on admission, and the uncontrolled blood glucose was an important risk factor for the exacerbation of the infection. The clinical manifestations of melioidosis are complex and varied, lacking specificity, making laboratory diagnosis difficult and the proportion of misdiagnosis and underdiagnosis is very high. The bacterium is usually not readily isolated from clinical specimens and, even if isolated, it may not be correctly identified. The patient was admitted with symptoms of respiratory tract infection, whose clinical manifestations and chest imaging were similar to that of tuberculosis. Initial impression was active lumbar tuberculosis with tuberculous spread, possibly with fungal super-infection. However, the blood culture results and MRI of the lumbar spine later showed that he had a B.pseudomallei infection and we considered that the multiple nodular shadows in his lungs could be migratory foci disseminated by bloodstream infection. For infection with complex history of the illness, notably bacteriology tests, and multiple lesions detected at imaging, integrative analysis is needed to make a diagnosis.

Treatment of melioidosis is biphasic and lengthy due to the rapid progression of the disease and the tendency of B.pseudomallei to cause latent infection. B.pseudomallei is extensively intrinsically resistant and requires long-term treatment, including an intensive intravenous phase and an oral eradication phase. The Ministry of Health Malaysia recommends carbapenems (imipenem or meropenem) or ceftazidime, alone or in combination with compound sulfamethoxazole as intensive therapy, and carbapenems are recommended in patients with severe sepsis, followed by an oral eradication phase lasting at least 12 weeks [6]. The presentation of urinary tract infection 5 months after the patient’s first discharge without formal eradication phase treatment was related to the experience of the physician and patient compliance. There is literature confirming that urinary tract melioidosis is a poorly known manifestation of the disease [7]. Since there was clear evidence of bacterial entry into the blood at the first admission, we considered urinary tract re-infection as secondary to the infection. For certain disease requiring long treatment times, following-up work is considered indispensable.

Acupuncture is an important traditional Chinese medicine treatment technique. It emphasizes the necessity for the operating physicianthat they are adequately trained in biomedical knowledge as well as in aseptic practice guidelines to minimise adverse acupuncture events. Acupuncture infection with B.pseudomallei is significantly rare in non-high prevalence areas. Due to deficiencies in the provision of medical history that patients may not associate acupuncture with the discomfort, there may be delays or errors in the diagnosis, while an inadequate course of treatment can lead to recurrence of the disease. Considering that bloodstream infections with B.pseudomallei caused by acupuncture have never been reported before, our case is an important reference for future cases similar to this one. It reminds the medical community to adhere to standard care sterile practice, while also emphasizing the importance of accurate diagnosis and adequate therapies.

## Data Availability

All data generated or analysed during this study are included in this published article.

## Abbreviations

MRI: Magnetic Resonance Imaging
MRSA: Methicillin-resistant *Staphylococcus aureus*
HBsAg: Hepatitis B surface antigen
HBeAb: Hepatitis B e antibody
HBcAb: Hepatitis B core antibody
CT: Computed Tomography
N: Neutrophil
HBA1c: Haemoglobin A1c
CRP: C-reactive protein

## References

[1] White A (2004). A cumulative review of the range and incidence of significant adverse events associated with acupuncture. Acupunct Med.

[2] Priola SM, Moghaddamjou A, Ku JC (2019). In reply to letter to the editor regarding “acupuncture-induced cranial epidural abscess: case report and review of the literature”. World Neurosurg.

[3] Limmathurotsakul D, Golding N, Dance D (2016). Predicted global distribution of Burkholderia pseudomallei and burden of melioidosis. Nat Microbiol.

[4] Wiersinga, W, Joost, et al. Melioidosis: insights into the pathogenicity of Burkholderia pseudomallei. Nat Rev Microbiol. 2006;4(4):272–82. 10.1038/nrmicro1385 16541135

[5] Wiersinga WJ, Virk HS, Torres AG, Currie BJ, Peacock SJ, Dance DAB, Limmathurotsakul D (2018). Melioidosis. Nat Rev Dis Primers.

[6] Sullivan RP, Marshall CS, Anstey NM, Ward L, Currie BJ (2020). 2020 review and revision of the 2015 Darwin melioidosis treatment guideline; paradigm drift not shift. PLoS Negl Trop Dis.

[7] Seppänen CP, Mikko. (2000). Melioidosis presenting as urinary tract infection in a previously healthy tourist. Scand J Infect Dis.

